# Metabolic impact of protein feeding prior to moderate-intensity treadmill exercise in a fasted state: a pilot study

**DOI:** 10.1186/s12970-018-0263-6

**Published:** 2018-11-29

**Authors:** Bradley T. Gieske, Richard A. Stecker, Charles R. Smith, Kyle E. Witherbee, Patrick S. Harty, Robert Wildman, Chad M. Kerksick

**Affiliations:** 1 Exercise and Performance Nutrition Laboratory, School of Health Sciences, Lindenwood University, St. Charles, MO 63301 USA; 2 Department of Exercise Science, Arnold School of Public Health, University of South Carolina, Columbia, USA; 3 Department of Food and Nutrition Sciences, Texas Woman’s University, Denton, TX USA

**Keywords:** Protein, Fasted exercise, Nutrient timing, Weight loss, Diet

## Abstract

**Background:**

Augmenting fat oxidation is a primary goal of fitness enthusiasts and individuals desiring to improve their body composition. Performing aerobic exercise while fasted continues to be a popular strategy to achieve this outcome, yet little research has examined how nutritional manipulations influence energy expenditure and/or fat oxidation during and after exercise. Initial research has indicated that pre-exercise protein feeding may facilitate fat oxidation while minimizing protein degradation during exercise, but more research is needed to determine if the source of protein further influences such outcomes.

**Methods:**

Eleven healthy, college-aged males (23.5 ± 2.1 years, 86.0 ± 15.6 kg, 184 ± 10.3 cm, 19.7 ± 4.4%fat) completed four testing sessions in a randomized, counter-balanced, crossover fashion after observing an 8–10 h fast. During each visit, baseline substrate oxidation and resting energy expenditure (REE) were assessed via indirect calorimetry. Participants ingested isovolumetric, solutions containing 25 g of whey protein isolate (WPI), 25 g of casein protein (CAS), 25 g of maltodextrin (MAL), or non-caloric control (CON). After 30 min, participants performed 30 min of treadmill exercise at 55–60% heart rate reserve. Substrate oxidation and energy expenditure were re-assessed during exercise and 15 min after exercise.

**Results:**

Delta scores comparing the change in REE were normalized to body mass and a significant group x time interaction (*p* = 0.002) was found. Post-hoc comparisons indicated the within-group changes in REE following consumption of WPI (3.41 ± 1.63 kcal/kg) and CAS (3.39 ± 0.82 kcal/kg) were significantly greater (*p* < 0.05) than following consumption of MAL (1.57 ± 0.99 kcal/kg) and tended to be greater than the non-caloric control group (2.00 ± 1.91 kcal/kg, *p* = 0.055 vs. WPI and *p* = 0.061 vs. CAS). Respiratory exchange ratio following consumption of WPI and CAS significantly decreased during the post exercise period while no change was observed for the other groups. Fat oxidation during exercise was calculated and increased in all groups throughout exercise. CAS was found to oxidize significantly more fat (*p* < 0.05) than WPI during minutes 10–15 (CAS: 2.28 ± 0.38 g; WPI: 1.7 ± 0.60 g) and 25–30 (CAS: 3.03 ± 0.55 g; WPI: 2.24 ± 0.50 g) of the exercise bout.

**Conclusions:**

Protein consumption before fasted moderate-intensity treadmill exercise significantly increased post-exercise energy expenditure compared to maltodextrin ingestion and tended to be greater than control. Post-exercise fat oxidation was improved following protein ingestion. Throughout exercise, fasting (control) did not yield more fat oxidation versus carbohydrate or protein, while casein protein allowed for more fat oxidation than whey. These results indicate rates of energy expenditure and fat oxidation can be modulated after CAS protein consumption prior to moderate-intensity cardiovascular exercise and that fasting did not lead to more fat oxidation during or after exercise.

## Background

Dietary fasting strategies have become increasingly popular in recent years to improve metabolic health, augment weight loss, and enhance body composition [1–3]. One common fasting strategy utilized by individuals seeking to maximize fat loss involves performing moderate intensity cardiovascular exercise following an overnight fast. Proponents of fasted cardiovascular exercise assert that the strategy increases rates of fat oxidation compared to postprandial exercise due to decreases in glycogen stores, low levels of circulating insulin, elevated lipolytic hormones and increased free fatty acid availability that characterize the post-absorptive state [4, 5]. Individuals also tend to extrapolate that an acute increase in fat oxidation translates to reductions in body fat over time when in fact, this outcome is multifaceted and more contingent upon energy balance changes over time than acute substrate utilization changes [6]. However, reports concerning the efficacy of post-absorptive exercise for facilitating weight loss and improving various metabolic health markers have been mixed. Chronic fasted endurance training has been shown to increase rates of both peripheral and intramyocellular fat oxidation [4, 7], upregulate maximal rates of oxidative enzyme activity [4], blunt intra-exercise glycogen breakdown [7], and improve both insulin sensitivity and glucose tolerance during a hypercaloric, high-fat diet compared to identical training conducted after feeding [8]. Furthermore, previous studies have demonstrated via whole-room indirect calorimetry that morning post-absorptive cardiovascular exercise resulted in greater accumulated fat oxidation across 24 h compared to intensity-matched postprandial exercise in both males [9, 10] and females during the early follicular phase of the menstrual cycle [11]. However, two recent training studies have reported similar rates of fat loss between females who performed either fasted or postprandial steady state (50 min at 70% heart rate reserve, 3 days per week) aerobic exercise [12] and high-intensity interval training [13] during extended periods of caloric restriction, thus reiterating the notion that caloric restriction is the primary contributor to fat loss rather than acute changes in fat oxidation.

One potential downside of post-absorptive cardiovascular exercise is the potential for breakfast to be delayed significantly or even skipped completely by exercising individuals. The consumption of high-protein morning meals has been shown to increase feelings of satiety during the day [14], reduce subsequent snacking behaviors [15], improve body composition [16], and potentiate weight loss in conjunction with a hypocaloric diet [17]. In addition, dietary protein exerts an anti-catabolic stimulus when ingested before or during exercise, providing a practical rationale for exercising individuals who may wish to minimize protein degradation during endurance exercise modalities [18]. Furthermore, preliminary evidence suggests that the acute ingestion of a high-protein meal immediately before exercise may have beneficial effects on post-exercise energy expenditure compared to pre-exercise carbohydrate ingestion [19, 20] or fasted conditions [5]. Indeed, research by Wingfield and investigators [20] used a crossover study design to examine the acute impact of protein or carbohydrate feedings prior to moderate aerobic exercise, high-intensity interval training, or resistance exercise sessions. When a single dose of whey protein was consumed before exercise, significantly greater increases in energy expenditure and fat oxidation were found to occur during the hour after exercise. Hackney and colleagues [19] noted that this effect of pre-exercise whey protein feeding on resting metabolism appears to last for at least 24 h after resistance exercise, though rates of fat oxidation were not different between carbohydrate or protein treatments. Likewise, Paoli et al. [5] reported that consuming a protein-rich meal prior to moderate-intensity cardiovascular exercise resulted in significant increases in resting metabolism for 24 h after exercise. While the meal induced acute elevations in RER relative to a control (fasted) condition, rates of fat oxidation were significantly lower at 12 and 24 h post-exercise in those who consumed a pre-exercise meal.

Clearly, targeted research must be conducted to further investigate the interaction between nutritional and exercise strategies which are purported to maximize fat loss. Given the demonstrated benefits of pre-exercise protein ingestion, performing cardiovascular exercise following a protein feeding may prove to be a more effective fat loss strategy than fasted exercise of similar intensity. Protein ingestion may improve short-term metabolic outcomes, as subtle elevations in RER due to protein intake may be offset by subsequent elevations in resting energy expenditure (REE) to increase the total quantity of fat oxidized during and after lower intensity, otherwise fasted exercise. However, as no investigation has evaluated the effect of different types of protein on metabolic outcomes during and after moderate-intensity aerobic exercise, it is relevant to examine whether different sources of protein differ in their effect on postprandial metabolism due to differing absorption kinetics and amino acid profiles [21]. Upon ingestion, whey protein passes quickly through the stomach and rapidly increases plasma amino acid levels, while casein gels and condenses in the stomach, resulting in delayed gastric emptying and a prolonged reduction in whole-body protein catabolism [22, 23]. Furthermore, whey and casein protein have been shown to differ greatly in their effect on postprandial metabolism, as the thermic effect of food (TEF) of a meal containing whey protein was found to be significantly greater than a similar meal containing casein [24]. Thus, the purpose of the current study was to quantify the effects of isocaloric and isonitrogenous pre-exercise feedings of whey protein isolate (WPI) and casein protein (CAS) on fat oxidation and energy expenditure during and after a bout of moderate-intensity treadmill exercise compared to isocaloric carbohydrate and control (fasted) conditions. It was hypothesized that pre-exercise protein ingestion would increase post-exercise energy expenditure and fat oxidation compared to both carbohydrate and fasting conditions. It was further hypothesized there would be no difference in energy expenditure and fat oxidation between the two sources of protein examined in this study.

## Methods

### Overview

This study was completed as a randomized, double-blind, placebo-controlled, crossover study design. All study participants completed four identical testing sessions. Participants completed all testing between 6:00–9:00 A.M. and all testing sessions for each participant were scheduled to begin at identical times. The order upon which all four conditions were completed was randomized using random allocation software. Prior to participation, all participants completed a familiarization session that consisted of providing their informed consent, determination of demographic information, submaximal exercise testing to determine heart rate prescription, and further orientation to the study protocol. Prior to each testing session, participants abstained from exercise for 24 h and observed a ten hour fast with only water ingestion being permitted during the fasting period. Prior to the first testing session, study participants completed a four-day dietary record that was copied and provided to all participants for them to replicate during the four days preceding each subsequent study visit. Participants were instructed to consume identical meals the evening before arriving for testing. Upon arrival, participants were weighed and completed a resting metabolic rate assessment over a 25-min time period using indirect calorimetry for determination of baseline rates of substrate oxidation and energy expenditure. Resting heart rate was determined upon completion of each initial resting metabolic rate assessment. Prior to exercise, participants were then randomized to ingest in a double-blind fashion one of four similarly colored and flavored isovolumetric (12 fluid ounces of cold water) solutions consisting of approximately 25 g of a whey protein isolate, 25 g of casein protein, 25 g of maltodextrin, or a non-caloric control. Participants then sat quietly for 30 min before completing a standardized warm-up protocol consisting of whole-body dynamic movements that lasted approximately ten minutes. Participants then completed 30 min of treadmill exercise at 55% heart rate reserve. Exercise heart rate was calculated by first predicting maximal heart rate (Max HR = 220 – age) and then adopting the methods of Karvonen et al. [25] to determine exercise heart rates. Determination of maximal aerobic capacity was not completed in this study as the intensity completed throughout the exercise bout was recorded minute by minute throughout the first testing visit and replicated for all subsequent testing sessions. Throughout each exercise bout, indirect calorimetry was continuously assessed while heart rates and ratings of perceived exertion (RPE) were assessed every minute. Upon completion of the exercise bout, each participant was provided with 12 fluid ounces of cold water and rested quietly. Approximately 15 min after completing the exercise bout, study participants then completed a second resting metabolic rate assessment using identical procedures. All metabolic rate assessments, supplement ingestion, warm-up, and treadmill exercise were directly supervised by a study investigator.

### Subjects

Eleven healthy, college-aged males (23.5 ± 2.1 years, 86.0 ± 15.6 kg, 184 ± 10.3 cm, 19.7 ± 4.4% fat) completed all four testing conditions. Participants were required to ingest no more than 300 mg of caffeine per day and abstained from any form of nutritional supplementation other than protein and multi-vitamins for 30 days prior to beginning the study protocol. All participants were recreationally active on most days of the week involving both endurance and resistance-based activities. None of the study participants were competitive athletes. All participants completed medical histories prior to participation and were excluded if they were currently diagnosed or being treated for any metabolic, renal, hepatic, cardiac, respiratory, musculoskeletal, or psychiatric disease. The study was approved by the Lindenwood University IRB (protocol # 861656–2, approval date: 3/4/2016), and all participants provided their written consent on an IRB-approved consent form prior to any data collection. Participants were recruited using flyers, social media, and word of mouth.

### Testing procedures

#### Demographics

Prior to their first study visit, participants had their standing height determined with their shoes removed while standing erect. Prior to each subsequent testing session, participants had their body mass determined on a Tanita model BWB-627A Class III digital scale *(Arlington Heights, IL)*. Resting heart rate values were then assessed for later calculation of exercise intensity.

#### Body composition

Body composition assessments were determined via dual-energy x-ray absorptiometry (DEXA) *(Hologic QDR Discovery A, Bedford, MA).* All participants underwent body composition assessment after observing at least an eight hour fast from all calorie-containing nutritional agents. In addition, participants refrained from physical activity for at least 24 h prior to the DEXA scan [26]. The machine was calibrated each day before any body composition testing and all scans were analyzed with the manufacturer-included software package *(Hologic APEX Software, Version 4.5.3)* using normative data derived from the 2008 National Health and Nutrition Examination Survey (NHANES) [27].

#### Dietary records

Dietary intake was assessed by having study participants complete a four-day food log that consisted of recording all food and fluid consumed over three weekdays and one weekend day prior to arriving for their first study visit. Each participant was instructed by a study team member on how to accurately complete a food record along with being provided multiple visual comparisons of certain foods to help with portion size estimation. All food records were analyzed by the same research team member using Vitabot online nutritional analysis software *(Vitabot, Riverdale, MD)*. All study participants returned a completed food record. The four-day food log was copied and provided to all participants for them to replicate during the four days preceding each subsequent study visit.

#### Supplementation protocol

In a randomized, double-blind, and crossover fashion participants were assigned to ingest one of four supplementation conditions: 25 g of a whey protein isolate (ISO100, Dymatize, Dallas, TX), 25 g of casein protein (ELITE Casein, Dymatize, Dallas, TX), 25 g of maltodextrin, or a non-caloric control. The maltodextrin and protein conditions were blinded by the manufacturer and the blinding codes were not revealed to research team members until completion of data collection. All drink solutions were similarly colored and flavored. Likewise, all test solutions were isovolumetric (12 fluid ounces of cold water), with the protein and carbohydrate beverages being isocaloric. Participants were given three minutes to consume their assigned supplement and upon ingestion were required to remain in a quiet room with low levels of stimulation for 30 min. During the last five minutes of the low-stimulation period, a standardized, dynamic warm-up consisting of whole-body dynamic movements was completed prior to beginning the treadmill exercise bout.

#### Resting measurements

All resting and exercising metabolic measures were completed using a ParvoMedics TrueOne 2400 metabolic measurement system *(ParvoMedics, Sandy, UT).* Each morning the indirect calorimetry system was calibrated by a research team member to ensure variations in measured oxygen and carbon dioxide and air flow rates were less than 2%. All subsequent tests were completed in an isolated, thermoneutral room with the lights illuminated. A blanket was provided and a clear plastic hood and drape was placed over each participant’s head and shoulders. The flow rate on the dilution pump was set to maintain approximately 0.8–1.2% carbon dioxide. Once an appropriate flow rate was established, study participants remained awake and motionless in a supine position for 20–25 min. The recorded data was visually inspected and a five-minute window where VO_2_ (in L/min) changed less than 5% was identified. From this group of data, resting energy expenditure values (in kcals/day) were calculated, and the average of all data points was computed.

#### Treadmill exercise protocols

All testing conditions were completed on a Woodway Desmo-Evo treadmill *(Woodway USA, Inc., Waukesha, WI USA).* During the familiarization session and prior to completing the testing conditions, all participants completed a graded, non-maximal exercise protocol to identify the approximate speed and grade combination that would elicit approximately 55% of each participant’s heart rate reserve (HRR) [25]. This protocol required each participant to walk for two minutes starting at a speed of 107.2 m/minute (4.0 mph) and 0% grade. Speed was then maintained at 107.2 m/minute while the grade was increased by 2% every two minutes until the observed heart rate values reached the desired heart rate. Each participant was outfitted with a Polar FT1 heart rate transmitter and chest strap *(Polar Electro Inc., Kempele, Finland).* Collected heart rates were recorded every minute, and the protocol was terminated when heart rate values equivalent to 55% of each participant’s heart rate reserve were achieved.

Once the desired speed and grade combination were determined, each participant then completed separate 30-min bouts of treadmill exercise at the individualized speed and grade combination that had been previously shown to elicit 55% of heart rate reserve. To match work completed across all four conditions, no changes in speed or grade were made throughout any portion of the completed exercise bouts. Indirect calorimetry was used to continuously assess oxygen consumption and substrate oxidation rates throughout each bout of exercise using a ParvoMedics TrueOne 2400 metabolic measurement system *(ParvoMedics, Sandy, UT).* On subsequent days, the metabolic cart was calibrated prior to testing following identical procedures. Heart rate was also continuously assessed using a Polar FT1 heart rate transmitter worn on the wrist and chest. Rating of perceived exertion (RPE) was assessed every minute on a 6–20 scale per the procedures of Borg [28]. Substrate oxidation rates (every five minutes) were calculated according to the methods of Weir et al. [29]. To further examine the effects of the nutritional interventions, the total fat oxidized during each five-minute period was calculated using standard thermal equivalents of oxygen [30].

### Statistical analysis

All data is presented as means ± standard deviations and was entered into Microsoft Excel (Seattle, WA USA) software and analyzed using IBM SPSS 23 (Armonk, NY USA). Energy expenditure data was normalized to body mass in kilograms. Data was first checked for normality using standardized skewness and kurtosis values. Log transformations were performed in cases where the assumption of normality was violated. However, all statistical outcomes did not change when using transformed data, therefore non-transformed data is presented throughout the paper. Mixed factorial ANOVAs (group x time) with repeated measures on time were used to assess the main effects for time and group as well as their interaction (group x time) for all outcome measures. A significance level of 0.05 was used to guide statistical decisions. A trend was decided a priori to be interpreted as any reported *p*-value that fell between *p* = 0.051–0.10. To fully decompose main and interaction effects, delta values were computed and graphs illustrating individual responses were computed in addition to calculating within-group effect sizes for each condition as well as the effect size of each nutrient condition (WPI, CAS, and MAL) in comparison to MAL and CON, respectively. All effect sizes were interpreted as small (d = < 0.2), moderate (d = 0.5), and large (d = 0.8) according to the methods of Cohen [31]. One-way ANOVAs with Tukey post-hoc comparisons were used to identify differences between testing conditions. Pearson correlations were computed to assess the presence of any relationships within the data. All results are presented as mean ± standard deviation.

## Results

### Dietary intake

The average four-day diet composition reported by participants prior to Visit 1 was as follows: 2446 ± 800 kcal (28.44 ± 9.30 kcal/kg), 132 ± 56 g (1.53 ± 0.65 g/kg) protein, 235 ± 101 g (2.73 ± 1.17 g/kg) carbohydrate, 99 ± 37 g (1.15 ± 0.43 g/kg) fat. Each participant was instructed to replicate this dietary intake across the remaining testing visits.

### Exercise intensity

One-way ANOVA revealed no significant differences (*p* = 0.743) in intra-exercise heart rate, rating of perceived exertion (*p* = 0.985), or oxygen consumption (*p* = 0.993) between conditions, suggesting that intensity was sufficiently standardized across all testing sessions.

### Energy expenditure

**Fig. 1:**
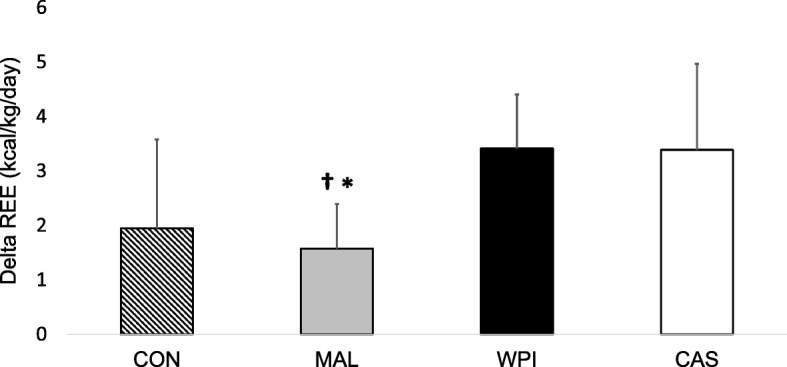
Delta scores (post-exercise minus pre-exercise of resting energy expenditure levels normalized to body mass in kg). WPI = Whey protein isolate; CAS = Casein; MAL = Maltodextrin; CON = Control. **†** denotes a significant (*p* < 0.05) difference between WPI and MAL. * denotes a significant (p < 0.05) difference between CAS and MAL

**Fig. 2:**
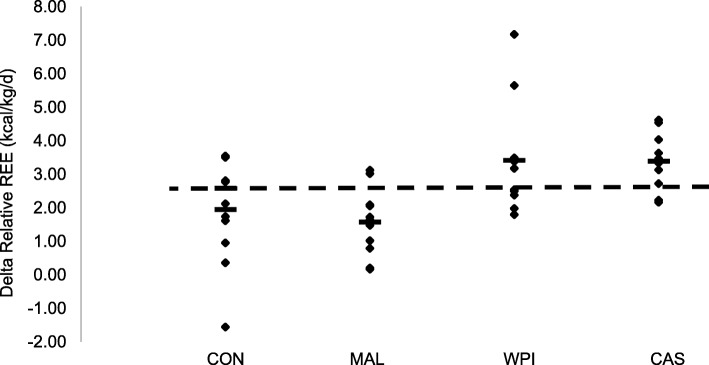
Individual responses of the delta value (post-exercise – baseline) in relative energy expenditure (kcals/kg/day). WPI = Whey protein isolate; CAS = Casein; MAL = Maltodextrin; CON = Control. Small black bars within each condition represents the average value for that experimental condition. Dotted line represents the grand mean for all four experimental conditions

Pre-treatment and pre-exercise rates of energy expenditure (Absolute: 1873 ± 189 kcal/day, Relative: 22 ± 2 kcal/kg/day) were not significantly different across conditions (*p* > 0.99). Rates of pre-exercise (pre-treatment) and post-exercise (post-treatment) resting energy expenditure (REE) were normalized to body mass (in kg) and a significant group x time interaction (*p* = 0.002) was found. To highlight the differences and changes across each exercise session, total estimated EE during exercise was quantified and delta scores were calculated by subtracting pre-treatment/exercise energy expenditure from post-exercise energy expenditure. One way ANOVA revealed significant differences between the delta scores (p = 0.002) and post-hoc comparisons indicated the within-group change in REE following consumption of WPI (3.41 ± 1.63 kcal/kg) was significantly greater (*p* < 0.05) than the within-group change in REE following consumption of MAL (1.57 ± 0.99 kcal/kg, *p* = 0.010) and tended to be greater than the non-feeding control group (2.00 ± 1.91 kcal/kg, *p* = 0.055). This trend is notable, as 73% of the participants during the WPI condition exhibited a change in REE toward the direction of significance. The within group change in REE following consumption of CAS (3.38 ± 0.82 kcal/kg) was greater than those following consumption of MAL (*p* = 0.012) and tended to be greater than the non-feeding control group (*p* = 0.061) (Fig. 1). Individual responses for each condition can be found in Fig. 2. A within-condition effect size for each nutrient (WPI, CAS, and MAL) was computed in addition to effect sizes comparing relevant nutrient responses to the changes seen in MAL and CON and can be found in Table 1. When compared to MAL and CON, the effect sizes for WPI and CAS were moderate to large (Table 1). Further, the number of participants during each condition that yielded a change in energy expenditure that was greater than the grand mean of all four conditions was greatest during CAS (9 out of 11 participants = 81.8%) followed by WPI (6 out of 11 participants = 54.5%), then MAL (2 out of 11 participants = 18.2%), and finally CON (5 out of 11 participants = 45.4%). One way ANOVA revealed that total estimated EE during exercise was significantly different between conditions (*p* = 0.002), and post-hoc comparisons showed that total intra-exercise EE was significantly higher (*p* < 0.05) after ingestion of WPI (345 ± 31 kcal), CAS (362 ± 32 kcal), and MAL (349.17 ± 70 kcal) when compared to CON (293 ± 37 kcal).

**Table 1:** Effect size calculations

Relative Energy Expenditure (kcal/kg/day)			
	Effect Size Within-Group	Effect Size (Nutrient vs. CON)	Effect Size (Nutrient vs. MAL)
WPI	1.15	0.91	1.36
CAS	1.32	1.15	2.00
MAL	0.63	− 0.28	–
CON	0.74	–	0.28
Respiratory Exchange Ratio			
	Effect Size Within-Group	Effect Size (Nutrient vs. CON)	Effect Size (Nutrient vs. MAL)
WPI	−0.77	−0.65	−0.97
CAS	−0.41	− 0.56	−1.04
MAL	0.04	0.04	–
CON	0.01	–	−0.04

*WPI* = Whey protein isolate; *CAS* = Casein; *MAL* = Maltodextrin; *CON* = Control. Effect size within-group = (post-exercise – pre-exercise) / pooled SD. Effect Size (Nutrient vs. CON) = Post-delta – Pre delta / pooled SD. Effect Size (Nutrient vs. MAL) = Post-delta – Pre-delta / pooled SD

### Substrate utilization

#### Post-exercise responses

**Fig. 3:**
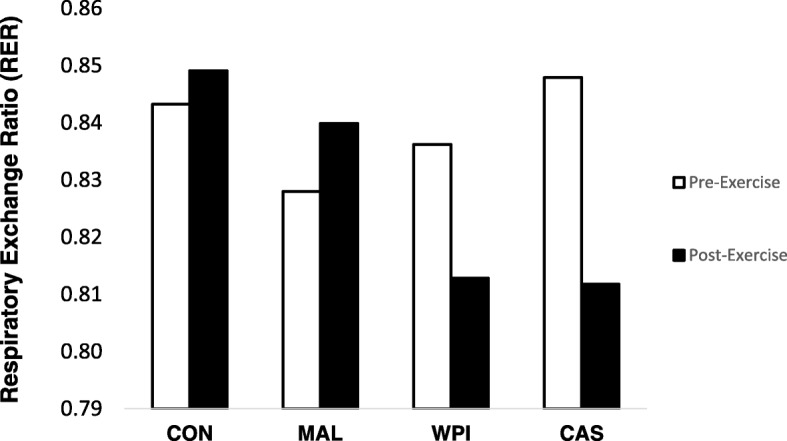
Changes in respiratory exchange ratio before and after exercise. Whey and casein significantly decreased from pre-exercise values in comparison to fasted control (p < 0.05)

**Fig. 4:**
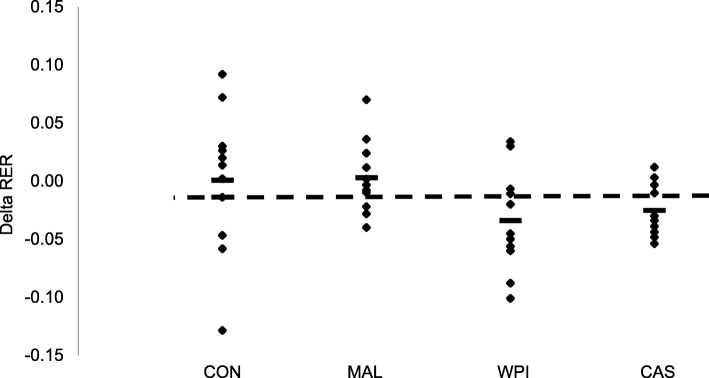
Individual responses of the delta value (post-exercise – baseline) in respiratory exchange ratio (RER). WPI = Whey protein isolate; CAS = Casein; MAL = Maltodextrin; CON = Control. Small black bars within each condition represents the average value for that experimental condition. Dotted line represents the grand mean for all four experimental conditions

No significant group x time interaction effect (*p* = 0.116) was found for respiratory exchange ratio (RER) data between pre-exercise and post-exercise resting metabolic rate measurements for all four experimental conditions (Fig. 3). To this end, RER significantly decreased (p < 0.05) from baseline following WPI (d = − 0.77) and CAS (d = − 0.41) consumption during the post-exercise measurement period while no such change (*p* > 0.05) was seen for the MAL (d = 0.04) or the non-feeding control groups (d = 0.01). No changes between WPI and CAS throughout the post-exercise measurements were noted. Individual responses (Fig. 4) and effect sizes for all changes seen in respiratory exchange ratio data were computed and can be found in Table 1. The number of participants during each condition that yielded a change in respiratory exchange ratio that was lower than all four conditions combined was similar during CAS (7 out of 11 participants = 81.8%) and WPI (7 out of 11 participants = 81.8%) when evaluated against MAL (3 out of 11 participants = 27.2%) and CON (3 out of 11 participants = 27.2%).

#### Intra-exercise responses

Intra-exercise (every five minutes) substrate oxidation rates were assessed and compared between feeding conditions. A main effect for time (*p* < 0.001) and a non-significant group x time interaction effect (*p* = 0.188) were identified for RER. Estimations of total fat oxidation were made for every 5-min time period through exercise. A significant main effect for time (p < 0.001) and a significant group x time interaction (*p* = 0.028) was found for total fat oxidation. To fully decompose the significant interaction effect, one-way ANOVAs were computed at each time point and revealed significant between-group differences in 5-min fat oxidation at 5–10, 10–15 min and 25–30 min of exercise (Fig. 6). Post-hoc follow-ups revealed that significantly more fat (*p* < 0.05) was oxidized after consumption of casein compared to WPI during minutes 10–15 (CAS: 2.28 ± 0.38 g; WPI: 1.7 ± 0.60 g) and 25–30 (CAS: 3.03 ± 0.55 g; WPI: 2.24 ± 0.50 g) of the exercise bout. Additionally, MAL consumption was found to oxidize greater amounts (p < 0.05) of fat in comparison to WPI during minutes 5–10 of exercise (MAL: 2.23 ± 0.42 g; WPI: 1.64 ± 0.68 g) (Fig. 6).

**Fig. 5:**
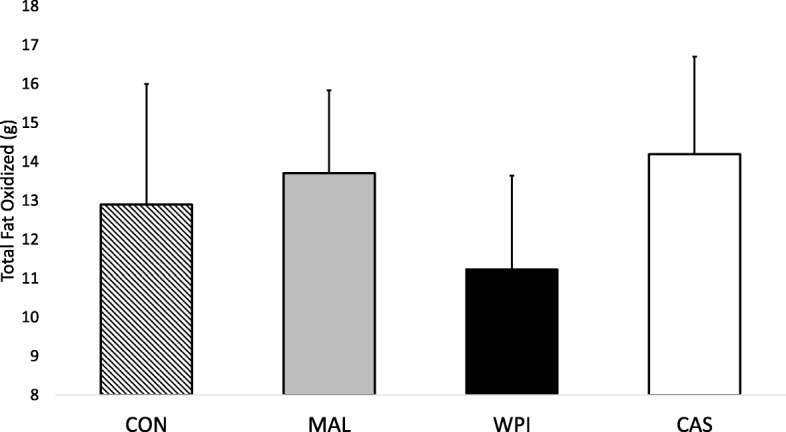
Estimated total fat oxidization throughout entire 30-min bout of moderate-intensity cardiovascular exercise. Values were calculated by multiplying each respective 5-min average of VO_2_ and RER against standard thermal equivalents and summed. Results were analyzed with One-way ANOVA. WPI = Whey protein isolate; CAS = Casein; MAL = Maltodextrin; CON = Control. * denotes a significant (p < 0.05) difference between WPI and CAS

**Fig. 6:**
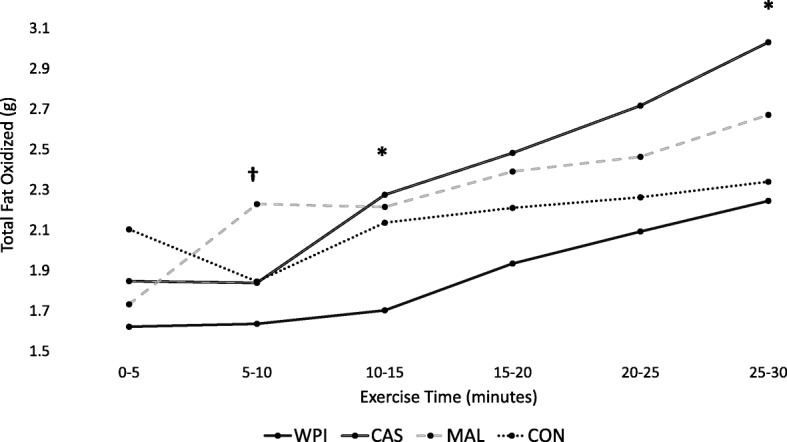
Total fat oxidized during each five-minute interval throughout completion of 30 min of moderate-intensity cardiovascular exercise. WPI = Whey protein isolate; CAS = Casein; MAL = Maltodextrin; CON = Control. **†** denotes a significant (p < 0.05) difference between WPI and MAL. * denotes a significant (p < 0.05) difference between WPI and CAS

## Discussion

The purpose of this investigation was to compare the effects of consuming supplemental levels of whey and casein, as well as carbohydrate, 30 min prior to a moderate intensity bout of treadmill exercise in comparison to completing an identical bout of exercise in a fasted state. The findings from this study indicate that exercising while fasted did not appreciably impact energy expenditure or substrate utilization either during or after exercise. Pre-exercise casein protein supplementation significantly increased rates of post-exercise fat oxidation and energy expenditure while whey protein resulted in less total fat oxidized during the exercise bout compared to casein (Fig. 5 and Fig. 6).

Results from the present study indicate that pre-exercise protein consumption (WPI: 15.9 ± 8.3% and CAS: 15.4 ± 3.5%) results in significant increases in resting energy expenditure following fasted moderate-intensity exercise compared to an isocaloric carbohydrate feeding (MAL: 7.3 ± 4.8%) or pre-exercise fasting (CON: 8.9 ± 6.7%). These findings align with the conclusions of similar investigations that evaluated the relationship between acute pre-exercise nutrition interventions and subsequent changes in post-exercise resting energy expenditure. Wingfield et al. [20] reported that an acute protein feeding resulted in significant elevations in resting energy expenditure for 60 min following exercise compared to a pre-exercise carbohydrate feeding. Such conclusions are supported by a well-developed body of research reporting that the consumption of high protein meals or short-term high protein diets results in elevated rates of postprandial dietary thermogenesis compared to lower-protein controls [24, 32–35]. Interestingly, a recent report has suggested that moderate intensity exercise may potentiate dietary thermogenesis. Kang et al. [36] reported that the TEF of a 721-kcal meal (23% PRO, 41% CHO, 36% FAT) consumed by subjects 60 min before moderate intensity exercise at 50% peak oxygen consumption (VO_2_peak) resulted in a two-fold increase in dietary thermogenesis compared to the isolated TEF of the meal while the subjects remained at rest. The results reported by Kang et al. [36] suggest that pre-exercise feeding significantly potentiates energy expenditure during exercise in both males and females, findings which support the conclusions of Davis and colleagues [37, 38]. Likewise, results reported by Stiegler et al. [39] support the notion that exercise-induced potentiation of dietary thermogenesis seems to only occur if exercise is performed after a meal. While such outcomes were not directly assessed by the design of the present study, such an effect would nonetheless align with the results of this study.

It is vital to mention that because an increase in resting energy expenditure was detected after every condition in the present study, a portion of the increased REE likely resulted from excess post-exercise oxygen consumption (EPOC) [40], particularly because of the close proximity that existed between cessation of the exercise bout and post-exercise REE measurements. However, Paoli et al. [5] highlighted in their discussion that an exercise bout consisting of 36 min of treadmill exercise at 65% HRR was not of sufficient intensity to result in appreciable EPOC after 12 h of recovery. Because the exercise intervention used in the present study was of similar duration (30 min) and intensity (~ 60% HRR), it is likely that EPOC played a relatively minor role in post-exercise metabolic changes. Similarly, the exercise intensity implemented in the present intervention and others falls within the range known to elicit maximal fat oxidation (45–65% maximal oxygen consumption (VO_2_max) [41]. Thus, the conclusions of this study regarding substrate utilization and energy expenditure should not be extrapolated to exercise interventions comprised of higher or lower exercise intensities or of durations that reach markedly beyond what was utilized in the present study.

The absence of differences in intra-exercise RER between conditions observed during this investigation somewhat contrasts with earlier reports which concluded that pre-exercise feeding blunts intra-exercise fat oxidation (Reviewed in Ref. [42]). However, differences in study duration, exercise intensity, timing of ingestion, amount of food and composition of food ingested, and training status of participants are all factors that may impact changes in energy expenditure and substrate oxidation. Regardless, one-way ANOVA revealed that total fat oxidized during several five-minute intervals of exercise was significantly lower after ingestion of WPI compared to CAS and MAL, potentially due to differences in absorption and insulin response between the two protein sources [43]. While this outcome was not directly measured in this investigation, it is possible that the insulin response to WPI ingestion in this investigation was greater than MAL, as Dalbo et al. [44] reported significant post-exercise elevations in insulin after pre-exercise ingestion of 25 g WPI but not MAL. While our work should certainly be considered preliminary and pilot in nature, these results suggest that casein protein may be preferable to whey protein with respect to intra-exercise fat oxidation. However, the augmented post-exercise reduction in RER following protein feeding observed during this investigation is in accordance with earlier studies and may be the result of transient elevations in protein synthesis [5, 20]. It is well-established that the relative contribution of lipids to metabolism increases during the recovery period following cessation of moderate intensity cardiovascular exercise (45–65% VO_2_peak) [45, 46]. In agreement with the present study, Wingfield and colleagues [20] observed a significant decrease in RER up to 60 min after exercise following a protein feeding compared to carbohydrate feeding, results which were corroborated by Paoli et al. [5], who noted a significant elevation in lipid utilization both 12 and 24 h after cessation of exercise completed in a postprandial state when compared to a post-absorptive state. However, these conclusions reached by Paoli et al. are not shared by Iwayama and colleagues [10, 11], who reported that 24-h rates of fat oxidation determined via metabolic chamber were greater in both males and females following a 60-min bout of post-absorptive cycling exercise at 50% VO_2_max compared to an identical bout of exercise performed after a standardized meal (15% PRO, 60% CHO, 25% FAT). It is important to note that the aforementioned studies primarily utilized mixed meals. Thus, the rates of digestion, TEF response, and fuel utilization likely varied greatly in comparison to the isolated nutrients provided in the current study.

Chronic relative macronutrient intake in the days prior to exercise appears to influence rates of substrate oxidation both during and after an exercise bout [20]. Patterson and Potteiger [47] compared substrate utilization kinetics between participants who consumed a low-carbohydrate, high-protein diet (40% PRO, 20% CHO, 40% FAT) or a moderate-carbohydrate diet (15% PRO, 55% CHO, 30% FAT) during the 48-h period before treadmill exercise at 55% VO_2_max. The researchers reported that the low-carbohydrate diet in conjunction with a two-hour pre-exercise fast elicited significantly increased rates of intra-exercise and post-exercise fat oxidation and significantly decreased rates of intra-exercise and post-exercise carbohydrate oxidation compared to the isocaloric, moderate-carbohydrate diet Because the dietary intake of the participants in the present study were not overtly controlled, but were advised to keep their nutrient intake the same prior to each visit, it is possible but not likely that any variation in dietary macronutrient ratios between conditions impacted our measured outcomes. In this respect, one should consider that all participants were required to complete a food record that was copied and replicated for each study for each subsequent study visit. Future research investigating metabolic outcome measures during and after exercise should ensure that all dietary intake is completely controlled in the days prior to testing visits.

Limitations of the current study include the lack of a mixed gender cohort and the absence of longer-duration metabolic assessment following the cessation of exercise, both of which reduce the generalizability of the study results. To completely assess the effect of pre-exercise feeding and protein source on post-exercise metabolism, future research should utilize intermittent follow-up metabolic measurements for at least 12 h following exercise, as inferences regarding long-term energy expenditure and substrate utilization cannot be adequately extrapolated from one acute post-exercise resting metabolic rate assessment. Finally, because no modifications were made to the participants’ self-directed pre-testing dietary intakes, substrate availability may have differed between participants and thus altered intra-exercise and post-exercise substrate utilization data. Future research in this area should implement a standardized diet prior to acute metabolic measurements to reduce any confounding influence of dietary intake.

## Conclusion

Results from this preliminary investigation suggest that consumption of 25 g of whey protein isolate or 25 g of casein protein 30 min before moderate-intensity treadmill exercise while fasted significantly increased rates of post-exercise energy expenditure when compared to the pre-exercise consumption of 25 g of maltodextrin or a non-caloric control. While differences in RER during exercise were not observed during either fasted cardiovascular exercise or post-prandial exercise, significantly more fat was oxidized following ingestion of casein vs. whey protein compared at two time points. Additional research is needed with longer exercise durations, varying exercise intensities, and nutrients consumed to better determine the impact of these findings.

## Abbreviations

ANOVA: Analysis of variance
CAS: Casein protein
CHO: Carbohydrate
CON: Control group
DEXA: Dual-energy x-ray absorptiometry
EPOC: Excess post-exercise oxygen consumption
FAT: Fat
HRR: Heart rate reserve
MAL: Maltodextrin
NHANES: National health and nutrition examination survey
PRO: Protein
REE: Resting energy expenditure
RER: Respiratory exchange ratio
RPE: Rating of perceived exertion
TEF: Thermic effect of food
VO_2_: Volume of oxygen consumption
VO_2_max: Maximal oxygen consumption
VO_2_peak: Peak oxygen consumption
WPI: Whey protein isolate
